# *RB1* screening of retinoblastoma patients in Sri Lanka using targeted next generation sequencing (NGS) and gene ratio analysis copy enumeration PCR (GRACE-PCR)

**DOI:** 10.1186/s12920-023-01721-6

**Published:** 2023-11-06

**Authors:** Nirosha Kugalingam, Deepthi De Silva, Hiranya Abeysekera, Sriyani Nanayakkara, Shamala Tirimanne, Dinali Ranaweera, Prashanth Suravajhala, Vishvanath Chandrasekharan

**Affiliations:** 1 Department of Chemistry, Faculty of Science, University of Colombo, Colombo, Sri Lanka; 2 Department of Physiology, Faculty of Medicine, University of Kelaniya, Kelaniya, Sri Lanka; 3 Lady Ridgeway Hospital, Colombo, Sri Lanka; 4 National Eye Hospital, Colombo, Sri Lanka; 5 Department of Plant Sciences, Faculty of Science, University of Colombo, Colombo, Sri Lanka; 6 Amrita School of Biotechnology, Amrita Vishwa Visyapeetham, Clappana, Kerala India; 7 Sri Lanka Institute of Biotechnology, Pitipana, Homagama, Sri Lanka

**Keywords:** Retinoblastoma, Targeted NGS, GRACE-PCR, *RB1*, Sri Lanka

## Abstract

**Background:**

Retinoblastoma (RB) a tumour affecting those under 5 years, has a prevalence of 1 in 20,000, with around twenty new diagnoses per year in Sri Lanka. Unilateral and bilateral RB presents around 24 and 15 months respectively. Approximately 10% are familial. Systematic genetic testing for germline pathogenic variants of *RB1*, the only gene associated with an inherited risk of RB, is unavailable in Sri Lanka. Genetic testing optimizes management of affected children and at-risk siblings. This study aimed to develop accessible genetic testing to identify children with a germline pathogenic variant of *RB1* in Sri Lanka.

**Methods:**

Targeted next generation sequencing (NGS) for detecting pathogenic sequence variants and Gene Ratio Analysis Copy Enumeration PCR (GRACE-PCR) for detecting *RB1* copy number variations (CNVs) were performed for 49 consecutive RB patients treated between 2016 and 2020 at the designated RB care unit, Lady Ridgway hospital, Colombo. Patients (bilateral RB (n = 18; 37%), unilateral n = 31) were recruited following ethical clearance and informed consent.

**Results:**

There were 26 (53%) females. Mean age at diagnosis was 18 months. Thirty-five patients (71%) had undergone enucleation. Germline pathogenic variants of *RB1* identified in 22/49 (45%) patients including 18 (37%; 12 bilateral and 6 unilateral) detected by targeted NGS (2 missense, 7 stop gained, 1 splice donor, 8 frameshift variants). Six were previously undescribed, likely pathogenic frameshift variants. Four bilateral RB patients had GRACE-PCR detected CNVs including one whole *RB1*, two intragenic deletions (exon 12/13; exon 11 and 23) and a partial duplication of exon 27. The only familial case (affected mother and child) shared the duplication. Only 2 of 4 CNVs and 10 of 18 pathogenic variants were confirmed by whole exome sequencing and Sanger sequencing respectively, due to funding limitations.

**Conclusions:**

The study identified pathogenic or likely pathogenic germline *RB1* sequence variants and copy number variants in 16/18 (89%) bilateral and 6/31(19%) unilateral cases, which is comparable to worldwide data (10–15% unilateral, 80–85% bilateral). Targeted NGS combined with GRACE-PCR significantly reduce the cost of *RB1* testing in Sri Lanka, and may widen access for genetic diagnosis of RB patients in other low and middle income countries.

**Supplementary Information:**

The online version contains supplementary material available at 10.1186/s12920-023-01721-6.

## Introduction

Retinoblastoma (RB) is a malignant tumour of the eye that develops in early childhood with more than 90% of cases presenting before the age of 5 years [1]. The estimated prevalence of this disease is around 1 in 15,000 to 1 in 20,000 live births [2] and in Sri Lanka, there are at least 20 new cases detected each year. Retinoblastoma develops from the immature cells of the retina [3]. When diagnosed and treated early, useful vision can be preserved and the eye can be salvaged with or without useful vision acuity and near normal quality of life of the child. When the diagnosis is delayed, removal of the eye [enucleation] is required and on rare occasions, life expectancy can be limited [4]. Unpublished data from Sri Lanka indicates that 75% of RB patients present at a late stage, with the majority requiring enucleations.

Retinoblastoma results from the inactivation of both copies of *RB1*, a gene located on chromosome 13q14.2 [5]. The functions of pRB include regulation of cell cycle arrest, apoptosis, cellular aging and differentiation [3, 6]. In sporadic RB affecting around 60% of patients, the inactivating *RB1* variants are localized to the retina, and are associated with a lower risk of a second tumour in the patient or risk of RB in their siblings [3]. In around 40% of RB cases, a germline (inherited) pathogenic variant is present in all cells, with the development of a second ‘hit’ in the remaining allele in the retina. Germline variants are associated with a higher risk of a second RB as well as higher risk of RB in the siblings and offspring [3, 7]. The age of onset of RB is younger in cases with germline pathogenic variants (mean ages at presentation of unilateral and bilateral RB are 24 and 12–15 months respectively). The majority (85%) of patients with bilateral and around 15% of unilateral RB have germline *RB1* pathogenic variants [1, 7]. The described mechanisms of inactivating *RB1* variants in the germline include nonsense (37%), frameshift (20%), splice site (21%), missense (5%), copy number variations of one or more exons or entire gene (15%) and rarely, involve the promoter region (1%) [8, 9].

Knowledge of the genetic status enables more effective management of the affected patient and their at-risk relatives, with better targeting of screening investigations using examination under anaesthesia (EUA) to those at high risk [10]. With better targeting of EUA to those at high risk with less frequent investigations of those at low risk, the costs of screening (including the costs of frequent hospital admissions, loss of daily wages for one or both parents and transport costs borne by the family) can be reduced. As a result, the financial costs to the families and the health service can be alleviated.

The use of genetic testing for detecting *RB1* pathogenic variants in low (LICs) and low-middle income countries (LMICs) is limited, most often due to costs of testing which are mainly borne by the family, as well as the lack of availability of accessible and cost-effective testing.

Next generation sequencing (NGS) is an efficient and widely used technique for the molecular diagnosis of multiple genetic disorders including cancer [11]. In this study we used targeted amplicon sequencing, which is as efficient, but less expensive, than the more commonly used hybrid capture method [12]. It is capable of detecting germline sequence variants including small indels but is unable to detect copy number variations (CNVs) caused by deletion of one or several exons of *RB1*.

Gene ratio analysis copy enumeration PCR (GRACE-PCR) is a recently described method to detect CNVs that can be used for amplicon sizes up to 200-250 bp [13]. We have used this approach to determine the possibility of using this as an alternative to the traditional hybrid capture NGS, or Sanger sequencing of the 27 exons of *RB1* and determination of CNVs using MLPA, which may not be easily available or affordable in LMICs. We believe that this is the first description of the use of GRACE-PCR for CNV analysis of *RB1*.

## Materials and methods

### Patient data and sample collection

Forty-nine consecutive patients with RB between 2016 and 2020, including 18 bilateral and 31 unilateral cases were recruited at the Lady Ridgeway hospital, the only designated hospital for RB care in Sri Lanka, following informed consent from the parent/caregiver. Ethical approval for the study was obtained from the ethics review board of the Lady Ridgeway hospital, Colombo. Sociodemographic data obtained at recruitment were recorded in a data sheet and later transferred to a computerized database. Both the paper and computer data were accessible only to the research team. Staging of the tumour, performed by the consultant ophthalmologist according to the IIRC (International Intraocular Retinoblastoma Classification) criteria was also recorded. Enucleated eyes had histopathological examination of the RBs using light microscopy for identification of the Flexner-Wintersteiner rosettes to differentiate these from other ocular tumours [14, 15]. Areas of viable tumours, necrosis and calcification were also recorded. An anticoagulated venous blood sample (1 ml) was collected and transported to the laboratory in sealed, sterile conditions for DNA extraction using QIAamp mini kit as per manufacturer’s instructions. The extracted DNA was quantified using a spectrophotometer. Whenever possible, samples were collected from the parents of patients.

### Targeted next generation sequencing

Lymphocyte DNA (200ng) from 49 RB patients were subjected to targeted next generation sequencing (Applied Biological Materials Inc, Canada) of the 27 exons and the promoter region of *RB1* (Accession no: NC_000013.11). Primers were designed to cover all coding sequences of *RB1*. The PCR generated amplicons were ligated with Illumina adapters, each of which had a unique index across the 49 samples. The fragments with adapters were enriched by PCR. All amplicons of 49 samples were mixed together for NGS on a NextSeq500 (2 × 75 bp PE), which were finally de-multiplexed based on the index-sample relationship to differentiate the samples using barcodes.

### Data analysis

Raw data (FastQ) of 49 samples were analysed using an in-house bioinformatics pipeline. The bioinformatics tools FastQC, BOWTIE 2, samtools, bcftools and vcftools were used to analyse the data. Quality of the raw data was checked using FastQC tool and data which passed the quality check were aligned with the human reference genome (Hg19) using BOWTIE2 tool. An in-house pipeline was used to analyse the data in addition to GATK tool from the service provider. The variant call format (VCF) file was analysed using variant effect predictor (VEP) tool and Illumina base space variant interpreter. Pathogenic variants were confirmed using dbSNP, ClinVar, COSMIC (Catalogue of somatic mutation in cancer), GnomAD (The Genome Aggregation Database) and HGMD (Human Gene Mutation Database). The pathogenicity of identified novel variants were determined using SIFT and PolyPhen scores which indicated probably damaging or deleterious and frameshift variants.

### Confirmation of pathogenic variants using Sanger sequencing

PCR amplification was carried out in a final volume of 25 μl containing 50 ng of template DNA, 0.2 mM dNTPs, 1.5 mM MgCl_2,_ 0.2 μM forward and reverse primers, 1x PCR buffer (Tris HCl, pH 8.3, 50 mM KCl) and 1U of *Taq* polymerase (UC Biotech). The following thermal cycling conditions were used: initial denaturation at 94 °C for 5 min, followed by, 35 cycles of 94 °C for 30 s, 50 °C − 64 °C for 30 s, and 72 °C for 30 s with a final extension at 72 °C for 10 min. Amplification of exon 1 required 5% of dimethyl sulfoxide (DMSO) and exon 14 and 17 required 2 mM MgCl_2_. The amplicons were sequenced at Macrogen, Inc. South Korea. Funding limitations enabled Sanger sequencing for confirmation of ten variants only.

### Gene ratio analysis copy enumeration PCR (GRACE-PCR)

DNA from the 31 patients with absence of a pathogenic sequence variant of *RB1* identified using targeted amplicon sequencing were subjected to GRACE-PCR. Primers were designed for 27 exons and promoter region of target gene (*RB1*) and reference gene (*CFTR*) to compare their copy numbers (Additional file 1). Primers were designed to have similar annealing temperatures and amplicon lengths. The melting temperature of amplicons (target and control region) were within 3 − 8 °C from each other. To increase the accuracy of this method, the analysis was performed during the exponential phase of PCR [13, 16].

PCR amplification was carried out in a final volume of 25 μl containing 50 ng of template DNA, 0.2 mM dNTPs, 1.5 mM MgCl_2,_ 0.2 μM forward and reverse primers, 1x PCR buffer (Tris HCl pH 8.3, 50 mM KCl), EvaGreen (1 μM) and 1U of *Taq* polymerase (UC Biotech). The following thermal cycling condition were used: initial denaturation at 94 °C for 5 min, followed by, 35 cycles of 94 °C for 30 s, 60 °C for 30 s, and 72 °C for 30 s with a final extension at 72 °C for 10 min. Number of cycles (exponential phase), annealing temperature and concentration of MgCl_2_ were optimized for each exon separately. Melting curve analysis was performed following PCR to detect the intensity of fluorescence in the temperature range 70 to 90 °C at a rate of 0.2 °C/s with the quantity of amplicon being represented by the peak height. The ratio of the peak heights of the melting curve of the target (*RB1*) to the control gene (*CFTR)* was calculated for patients and normal controls separately. A ratio of patient to normal of 1 indicated absence of a deletion, a ratio 0.5 indicated presence of a deletion and a ratio of 1.5 indicated a duplication [14, 18].

### Whole exome sequencing (WES)

Extracted DNA was quantified using a nanodrop spectrophotometer. Whole exome sequencing was performed at Macrogen, South Korea. Paired-end sequencing was performed with 100X coverage using 1.0 µg of genomic DNA. Copy number variation (CNV) analysis was performed at Credence Genomics Pvt, Sri Lanka. CNVs in chromosomes were detected using read count data from recalibrated BAM files using ExomeDepth tool (a CNV calling algorithm with literature evidence on calling CNVs with relatively high accuracy using WES data) and CNVkit (a genome-wide copy number detection tool from high throughput sequencing), and the results of the two CNV tools were compared. Limitation of funding enabled the confirmation of 2 CNVs only.

### Family studies

The available genomic DNA of the parents of nine cases with an identified germline pathogenic variant were used to PCR amplify and then Sanger sequence exons containing these variants. PCR amplification was carried out essentially as described previously. One RB affected mother of a child identified to have a partial duplication of exon 27 of *RB1* was included for GRACE-PCR analysis.

## Results

### Clinical findings

There were 26 females (53%) among RB affected cases. The mean age at diagnosis of RB was 18 months. Unilateral tumours were present in 31 patients (63%) who had a mean age at diagnosis of 24 months. Bilateral tumours were present in 18 patients whose mean age at first diagnosis was 9 months. The IIRC stage at diagnosis for the 67 eyes were: group A − 6 eyes (9%), group B – 10 eyes (15%), group D − 24 eyes (36%) and group E − 27 eyes (40%). Thirty-five patients (71%) had undergone enucleation. The most common presenting features were leukocoria (white pupil) and strabismus (squint). Twenty-four patients had leukocoria, eight had squint while fourteen had both clinical features. Reddish discolouration of the eye has been noted in a few patients in addition to either leukocoria or squint (Table 1). One familial case (affected mother with bilateral RB requiring bilateral enucleations; child with bilateral RB diagnosed at 2 months and treated with enucleation of one eye and successful ocular salvage for the second eye) was included in this study.

**Table 1:** Clinical features of 49 patients

No.	Clinical Features	Frequency
**01.**	**Laterality (n = 49)**	
	Unilateral	31 (63%)
	Bilateral	18 (37%)
**02.**	**Gender (n = 49)**	
	Male	23 (47%)
	Female	26 (53%)
**03.**	**Mean age at diagnosis**	18 months
	Unilateral	24 months
	Bilateral	9 months
**04.**	**IIRC Classification (n = 67 eyes)**	
	Group A	06 (09%)
	Group B	10 (15%)
	Group C	00 (00%)
	Group D	24 (36%)
	Group E	27 (40%)
**05.**	**Familial RB**	01 (02%)
**06.**	**Symptoms at first presentation (n = 49)**	
	Leukocoria	24 (49%)
	Squint	08 (16%)
	Leukocoria and Squint	14 (29%)
	Others	03 (06%)

### Histopathological findings

Seven tumours were well differentiated, twelve were moderately differentiated and two were poorly differentiated RB. One tumour, a moderately differentiated RB had optic nerve involvement. Five tumours showed extensive necrosis, three showed regressed RB and two showed calcifications. Three cases had uveal tract involvement, gliosis with evidence of haemorrhage and vitreous seeding (Additional file 2).

### Targeted next generation sequencing

Pathogenic variants (2 missense, 7 stop gain, 1 splice donor, 8 frameshift variants) identified in 18/49 (37%) cases including 12/18 (67%) bilateral cases and 6/31(19%) unilateral cases. Six were previously undescribed, likely pathogenic frameshift variants (Table 2).

**Table 2:** RB1 pathogenic variants identified by targeted NGS

Patient ID	Nucleotide change	Amino acid change	Consequences	Confirmed by Sanger sequencing	Family study
RB 7	c.1759G > T	p.Glu587X	Stop gained	Yes	No
RB 8	c.2239G > T	p.Glu747X	Stop gained	No	No
RB 10*	c.1434dup	p.Asp479X	Frame shift	No	No
RB 11	c.137G > A	p.Arg46Lys	Missense	No	No
RB 14	c.2359_2360insT	p.Arg787LeufsX8	Frame shift	No	No
RB 15	c.1363 C > T	p.Arg455X	Stop gained	Yes	Yes
RB 17	c.2513 C > G	p.Ser838X	Stop gained	Yes	Yes
RB 21	c.1960G > T	p.Val654Leu	Missense	Yes	Yes
RB 26*	c.1865del	p.Val622GlufsX21	Frame shift	No	No
RB 30*	c.1672del	p.Met558TrpfsX53	Frame shift	No	No
RB 34	c.1333 C > T	p.Arg445X	Stop gained	Yes	Yes
RB 35*	c.1269del	p.Tyr424ThrfsX33	Frame shift	Yes	Yes
RB 37*	c.393_400del	p.Phe132ThrfsX5	Frame shift	Yes	Yes
RB 44	c.1735 C > T	p.Arg579X	Stop gained	Yes	Yes
RB 45*	c.518_519del	p.Tyr173PhefsX11	Frame shift	No	No
RB 46	c.83del	p.Pro28LeufsX37	Frame shift	Yes	Yes
RB 48	c.1633G > T	p.Glu545X	Stop gained	No	No
RB 49	c.380 + 1G > A		Splice donor	Yes	Yes

*Novel variants (Table 1) identified in this study

Pathogenic variants were identified using the following criteria (Table 3). (Devarajan et al., 2015)

1. *Known pathogenic variants are identified using a mutation database (e.g. dbSNP, ClinVar, HGMD (Human genome mutation database))*
2. *Variants giving rise to premature termination codons, frameshift, splice variant and large insertion or deletions.*
3. *Non-synonymous variants identified to determine pathogenicity (using for example SIFT, Polyphen)*

Identified pathogenic variants were confirmed using Sanger sequencing.

**Table 3:** Twelve *RB1* pathogenic variants identified in available databases

Patient ID	dbSNP	COSMIC	ClinVar	HGMD	SIFT	Polyphen
RB 7	LOVD Database				Deleterious	Possibly damaging
RB 8		COSM212665			Deleterious	Possibly damaging
RB 11		COSM5685202		CM092253	Unknown	Unknown
RB 14			Pathogenic		Deleterious	Possibly damaging
RB 15	rs121913302	COSM1367229 COSM895	Pathogenic	CM961228	Deleterious	Possibly damaging
RB 17		COSM5781553	Pathogenic	CM952423	Deleterious	Possibly damaging
RB 21	rs483352690	COSM2150876	Pathogenic	CM034902	Deleterious	Deleterious
RB 34	rs3092891	COSM1367224 COSM880	Pathogenic	CM900192	Deleterious	Possibly damaging
RB 44	rs121913305	COSM1756816 COSM892	Pathogenic /Likely Pathogenic	CM941206	Deleterious	Possibly damaging
RB 46	rs1276653645				Deleterious	Possibly damaging
RB 48	rs754354560	COSM947795		CM040262	Deleterious	Possibly damaging
RB 49		COSM1017	Pathogenic		Deleterious	Possibly damaging

Six germline pathogenic variants identified in our study which are novel, are likely pathogenic frameshift variants. These variants are predicted as deleterious in SIFT and possibly damaging in Polyphen databases.

### GRACE-PCR

Thirty-one DNA samples from RB patients without an identified pathogenic variant on targeted amplicon sequencing (25 unilateral, mean age at presentation, 23 months; 6 bilateral, mean age at presentation, 6 months) were included for this part of the study. Gradient PCR was carried out for each exon (27 exons and promoter region) and annealing temperatures were optimized. The concentration of MgCl_2_ and exponential phase of PCR were optimized for each primer set.

**Fig. 1:**
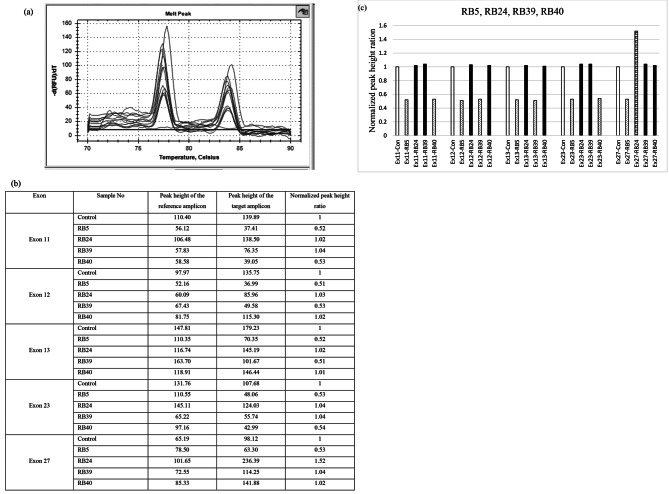
GRACE-PCR results of CNVs in patients RB 5, RB 24, RB 39 and RB 40. **a**. Graph shows the melting curves of patients and control after 23rd cycle. **b**. Peak height ratio calculation. **c**. Column chart results. White column represents wild type, dotted column represents patient with a deletion, hatched column represents patient with a duplication and black columns represent patients without a deletion or duplication. RB5 had a whole gene deletion and only exons 11, 12, 13, 23 and 27 are shown in this figure. RB 24 had a partial duplication of Exon 27. Exon 27 of *RB1* is 1893 bp in size, and its amplification required three primer pairs to analyse for CNVs in this exon. The graph shows results from one primer pair of exon 27. (Con, Control; Ex, Exon; RB, Patient ID)

There were three (3) deletions identified. One case (RB5) involved a deletion affecting all exons and promoter region (whole gene), one (RB39) had deletions of exons 12 and 13 and one (RB40) had deletions of exons 11 and 23. A child (RB24) whose mother was also affected had a partial duplication of exon 27, which was also identified in the mother (Fig. 1). WES confirmed the GRACE-PCR detected deletion in RB5 and duplication in RB24.

Total cost (consumables, chemical and reagents) for targeted amplicon sequencing plus GRACE-PCR per sample was around Rs. 25,000 (USD 133). Turnaround time of this diagnostic method was 8–10 weeks when targeted amplicon sequencing and GRACE-PCR were performed simultaneously.

### Family study

There were no germline pathogenic variants identified in nine unaffected parent pairs. In the single familial case with RB, both the affected mother and child shared the partial exon 27 duplication identified by GRACE-PCR and this was confirmed by WES.

## Discussion

The majority (71%) of Sri Lankan patients with RB presented with group D and E tumours and the majority of these children required enucleation as the main modality of treatment, at these later stages of RB. Genetic testing enables accurate identification of patients with an inherited predisposition to RB and will enable more intensive screening for and early diagnosis of a second tumour. Early diagnosis will prevent the need for enucleation and resulting blindness. Similarly, at-risk first degree relatives can also be identified early for screening investigations. Genetic testing is not currently available for the majority of patients with RB in Sri Lanka. Although the health service offers free healthcare, it does not fund molecular diagnostic testing and the patients’ parents are usually unable to afford the costs for these tests. The intention of this study was to develop molecular diagnosis for *RB1* pathogenic variants that is cost effective and applicable in Sri Lanka and similar LMICs with limited resources for molecular diagnosis.

Targeted amplicon sequencing and GRACE-PCR were performed in this study and, to the best of our knowledge, this is the first time that GRACE-PCR has been used to identify germline pathogenic CNVs in *RB1.*

There are two common techniques for targeted next generation sequencing, namely amplicon sequencing (using multiplex PCR) and hybrid capture based target enrichment. Amplicon sequencing requires lower input of DNA, has shorter and easier workflow, better performance on difficult clinical samples such as paraffin embedded tumour tissue DNA and is less expensive. Limitations of this method include the high, non-specific PCR background noise and need to design a large number of multiplex PCR primers. The biggest disadvantage is its inability to detect CNVs because PCR amplifies the wild type copy with the final amplicon not being proportional to the DNA content. Therefore, in *RB1* testing, a separate method of CNV analysis is required.

This study opted to use GRACE-PCR for CNV analysis. This investigation is unaffected by concentration of the template DNA, since both reference and target fragments are amplified in one tube. Results are unaffected by primer related artefacts since analysis of fragments depends on the unique melting temperature of each amplicon. The clear advantage of GRACE-PCR is that this method uses equipment that is usually available in molecular biology laboratories even in LMICs and analysis of GRACE-PCR is also relatively simple.

This study identified eighteen pathogenic *RB1* variants (2 missense, 7 stop gain, 1 splice donor, 8 frameshift variants) using targeted amplicon sequencing among 49 (37%) RB patients. Using GRACE-PCR for identification of CNVs, three intragenic deletions and one familial duplication were identified. The study identified *RB1* pathogenic variants in 22/49 (45%) cases including 16/18 (89%) bilateral cases and 6/31(19%) unilateral cases. The data we report here is similar to previously reported data from other countries, where germline pathogenic variants were identified among 80–85% of bilateral and 10–15% of unilateral RB patients [1, 7]. Two CNVs detected by GRACE-PCR (deletion of a whole gene of RB5 and partial duplication of exon 27 of RB24) were confirmed by WES.

In one familial case, the index patient had a partial duplication of exon 27, and this was also identified in the affected mother. This study was only able to test nine pairs of parents of children in whom a germline pathogenic variant was identified, and none of these clinically unaffected parents shared the variant with their affected child. In about 10% of RB cases, a germline pathogenic variant is identified in a parent, with the remainder expected to be due to pathogenic *de novo* variants or presence of somatic or gonadal mosaicism in a parent [7, 17].

The combination of amplicon sequencing and GRACE-PCR is a cost-effective method compared to other currently available tests including WES, panel based next generation sequencing and exon by exon Sanger sequencing with CNVs detected using QM-PCR or MLPA (the latter is not widely available locally). In a recent study, pathogenic variants were identified using the *RB1* gene capture approach, while significant deletions and duplications were identified using MLPA [18]. This approach however is more expensive than the amplicon sequencing and GRACE PCR used in this study.

## Conclusion

Currently, most if not all RB patients are managed without the use of genetic testing to guide management in Sri Lanka. Although genetic testing is available, the costs are often prohibitive for families who need to pay for the test. This study successfully developed a method of reducing the cost of genetic testing to make it more accessible for Sri Lankan patients. It may optimize the future management of RB affected Sri Lankan patients and their families.

### Electronic supplementary material

Below is the link to the electronic supplementary material.

**Additional file 1**: *RB1* (target gene) primers and *CFTR* (control gene) primers for GRACE-PCR

**Additional file 2**: Characteristics of RB patients selected for this study

## Data Availability

The datasets used and/or analysed during the current study are available from the corresponding author on reasonable request. The wild type *RB1* gene used for analysis in the current study is available in NCBI [RB1 RB transcriptional corepressor 1 [Homo sapiens (human)] - Gene - NCBI (nih.gov)]. Variants were identified in the study using the following databases: dbSNP, ClinVar, COSMIC (Search results for RB1 (sanger.ac.uk)), GnomAD (RB1 | gnomAD v2.1.1 | gnomAD (gnomad-sg.org)), LOVD (Transcript #00017482 (NM_000321.2, RB1 gene) - Global Variome shared LOVD) and HGMD (HGMD® home page (cf.ac.uk)).

## List of abbreviations

BL: Bilateral
*CFTR*: Cystic fibrosis transmembrane conductance regulator
CNVs: Copy number variations
COSMIC: Catalogue of somatic mutation in cancer
dbSNP: Single Nucleotide Polymorphism database
DMSO: Dimethyl sulfoxide
DNA: Deoxyribonucleic acid
dNTPs: Deoxyribonucleotide triphosphates
EUA: Examination under anaesthesia
GnomAD: Genome Aggregation Database
GRACE-PCR: Gene ratio analysis copy enumeration PCR
HCl: Hydrochloric acid
HGMD: Human Gene Mutation Database
IIRC: International Intraocular Retinoblastoma Classification
KCl: Potassium chloride
LICs: Low income countries
LMICs: Low-middle income countries
MgCl_2_: Magnesium chloride
MLPA: Multiplex ligation-dependent probe amplification
NGS: Next generation sequencing
PCR: Polymerase chain reaction
PE: Paired end
pRB: Retinoblastoma protein
QM-PCR: Quantitative multiplex polymerase chain reaction
*RB1*: RB Transcriptional Corepressor 1
RB: Retinoblastoma
SIFT: Sorting intolerant from tolerant
UL: Unilateral
VCF: Variant call format
VEP: Variant effect predictor
WES: Whole exome sequencing
