# Supplementary material for: RB1 screening of retinoblastoma patients in Sri Lanka using targeted next generation sequencing (NGS) and gene ratio analysis copy enumeration PCR (GRACE-PCR)
Source: BMC Med Genomics. 2023 Nov 6;16:279. doi: 10.1186/s12920-023-01721-6 (PMC10626775; doi:10.1186/s12920-023-01721-6)
Supplement: Supplementary file 1 — Additional file 1: RB1 (target gene) primers and CFTR (control gene) primers for GRACE-PCR [file 12920_2023_1721_MOESM1_ESM.docx]

**Additional file 1.** *RB1* (target gene) primers and *CFTR* (control gene) primers for GRACE-PCR

*RB1* (target gene) primers for GRACE-PCR.

| Exons | Primer ID | Sequence | Size (bp) | Tm of Primer (°C) | % GC | Amplicon size (bp) | Tm of amplicon (°C) |
| --- | --- | --- | --- | --- | --- | --- | --- |
| Promoter | ProFP | CCCAGTTTAATTCCTCATGACTTAGC | 26 | 62.6 | 42.3 | 223 | 71.9 |
|  | ProRP | TCTCCCGACTCCCGTTACA | 19 | 63 | 57.9 |  |  |
| 1 | HRMEx1F | TGTAACGGGAGTCGGGAGA | 19 | 63 | 58 | 208 | 70.8 |
|  | HRMEx1R | TGCTCTGGGTCCTCCTCA | 18 | 63 | 61 |  |  |
| 2 | HRMEx2F | TGGTAGGCTTGAGTTTGAAGA | 21 | 60.4 | 42.9 | 264 | 84.8 |
|  | HRMEx2R | TCTGGGTAATGGAATTATTATTAGCT | 26 | 59.7 | 30.8 |  |  |
| 3 | HRMEx3F | ACTGTGGGGAATCTGTATCTTTAT | 24 | 60.7 | 37.5 | 255 | 82 |
|  | HRMEx3R | TCATAACGGCTCCATGAGAGA | 21 | 61.6 | 47.6 |  |  |
| 4 | HRMEx4F | TAGAGGTGTAAGTTGAAGGCTAA | 23 | 60.1 | 39.1 | 236 | 84.6 |
|  | HRMEx4R | CATTATCAACTTTGGTACTGGTATC | 25 | 58.9 | 36 |  |  |
| 5 | HRMEx5F | AGGACATGTGAACTTATATATTTGACA | 27 | 60.3 | 29.6 | 243 | 83.7 |
|  | HRMEx5R | TCTTTGTAGTACAAGGCATGTAT | 23 | 59 | 34.8 |  |  |
| 6 | HRMEx6F | GATATCTACTGAAATAAATTCTGCAT | 26 | 57.4 | 26.9 | 255 | 84.5 |
|  | HRMEx6R | TAAATAAAGCATTTCCAAGGTTG | 23 | 57.4 | 30.4 |  |  |
| 7 | HRMEx7F | AGGGGAAGTATTACAAATGGAAGAT | 25 | 61.3 | 36 | 256 | 81.9 |
|  | HRMEx7R | ACTTACACTACTTATGGGCAAAGTC | 25 | 61.8 | 40 |  |  |
| 8 | HRMEx8F | ATTGTTATCCTTCTAATGAAACCTA | 25 | 57.8 | 28 | 261 | 83.1 |
|  | HRMEx8R | TACAGAGAACTTCAATAATTCTTGTATCA | 29 | 60.7 | 27.6 |  |  |
| 9 | HRMEx9F | TTATACCTTTTATGAATTCTCTTGGAC | 27 | 59.2 | 29.6 | 194 | 81.8 |
|  | HRMEx9R | ACCACAATTCTACTTGGCTAGA | 22 | 60.3 | 40.9 |  |  |
| 10 | HRMEx10F | ATGCGAACTCAGTGTATATTACA | 23 | 59 | 34.8 | 242 | 84.2 |
|  | HRMEx10R | CTGTCTATAGAATCAGTCTGAAGAGT | 26 | 60.5 | 38.5 |  |  |
| 11 | HRMEx11F | AGCTGGGTCATCTATTTTCTATCCTA | 26 | 62.2 | 38.5 | 230 | 82.8 |
|  | HRMEx11R | CTAACTGGAGTGTGTGGAGGA | 21 | 61.9 | 52.4 |  |  |
| 12 | HRMEx12F | AGGACTGTTATGAACACTATCCA | 23 | 60.2 | 39.1 | 210 | 83.5 |
|  | HRMEx12R | ATGGATAACTACATGTTAGATAGGAGA | 27 | 60.4 | 33.3 |  |  |
| 13 | HRMEx13F | AGAACTGCACAGTGAATCCA | 20 | 60.6 | 45 | 252 | 82 |
|  | HRMEx13R | AGCAGGGATATAGTATCTGACAGT | 24 | 61.4 | 41.7 |  |  |
| 14 | HRMEx14F | TAGCGATACAAACTTGGAGTTC | 22 | 59.3 | 40.9 | 259 | 77.2 |
|  | HRMEx14R | TAGCTGGGACTACAGGTCTA | 20 | 59.9 | 50 |  |  |
| 15 | HRMEx15F | AGACCTGTAGTCCCAGCTA | 19 | 60.1 | 52.6 | 245 | 81.5 |
|  | HRMEx15R | TGAATGGATAATCGTTCTTCTTCCT | 25 | 61.4 | 36 |  |  |
| 16 | HRMEx16F | AGCAAACTTCTGAATGACAACA | 22 | 60.2 | 36.4 | 238 | 82.3 |
|  | HRMEx16R | TGGCTTATAATGACCAATTACATTCT | 26 | 60.5 | 30.8 |  |  |
| 17 | HRMEx17F | AGGAAGTACATCTCAGAATCTTGA | 24 | 60.3 | 37.5 | 249 | 82.6 |
|  | HRMEx17R | TGTTAGCCATATGCACATGAAT | 22 | 59.7 | 36.4 |  |  |
| 18 | HRMEx18F | ACAATCAAAGGACCGAGAAGGA | 22 | 62.6 | 45.5 | 259 | 83.3 |
|  | HRMEx18R | TGACTTTATTTGGGTCATGTACCT | 24 | 61.3 | 37.5 |  |  |
| 19 | HRMEx19F | TATCTTTCTCCTGTAAGATCTCCAA | 25 | 60.2 | 36 | 225 | 80.6 |
|  | HRMEx19R | TAGAAAGTAGAAGAAACATGATTTGAAC | 28 | 60 | 28.6 |  |  |
| 20 | HRMEx20F | AGAGTGGTAGAAAAGAGGTTTCTGTTA | 27 | 63 | 37 | 251 | 81.4 |
|  | HRMEx20R | ATACTCATTCTGCAGGGTGTGCT | 23 | 64.9 | 47.8 |  |  |
| 21 | HRMEx21F | TGATGTGTTCCATGTATGGCA | 21 | 61.1 | 42.9 | 231 | 83.2 |
|  | HRMEx21R | TGTGAATTTACATAATAAGGTCAGACA | 27 | 60.5 | 29.6 |  |  |
| 22 | HRMEx22F | TGTGCTTCTTACCAGTCAAAAAG | 23 | 60.6 | 39.1 | 249 | 85.5 |
|  | HRMEx22R | TACCCTGGTGGAAGCATACTG | 21 | 62.3 | 52.4 |  |  |
| 23 | HRMEx23F | TACCTTGTCACCAATACCTCA | 21 | 59.6 | 42.9 | 261 | 80.5 |
|  | HRMEx23R | GTCTAGCTTATTTGAAATGAAGAATG | 28 | 61.9 | 32.1 |  |  |
| 24 | HRMEx24F | ATCTTAGTATCAATTGGTGAATCATTC | 27 | 59.5 | 29.6 | 239 | 84.4 |
|  | HRMEx24R | GTTTTGAAGTTCACCAATTAGGAGT | 25 | 61.1 | 36 |  |  |
| 25 | HRMEx25F | AGTTATTACCTTTGCCTGATTTTTGA | 26 | 61.2 | 30.8 | 239 | 81.7 |
|  | HRMEx25R | ATCTGATCCTTCAATATCAAAGCGT | 25 | 61.9 | 36 |  |  |
| 26 | HRMEx26F | ATCTGCAGTAAACATCTCCCA | 21 | 60.3 | 42.9 | 236 | 84 |
|  | HRMEx26R | ACATCATACATTTAATCCACAAATGT | 26 | 59.6 | 26.9 |  |  |
| 27 | HRMEx27F | ACTGTGTACACCTCTGGATTC | 21 | 60.3 | 47.6 | 245 | 82.2 |
|  | HRMEx27R | TTACTTTGGAAGAGGAAACAATCT | 24 | 59.8 | 33.3 |  |  |
|  | HRMEx27F1 | ACTATTGGAATCTGATATACTGTGTG | 26 | 59.8 | 34.6 | 201 | 82.9 |
|  | HRMEx27R1 | ACCAAGAGTACTATCAATAATCCTCA | 26 | 60.6 | 34.6 |  |  |
|  | HRMEx27F2 | ATCTCAAAATTATTCTGCCCTCCT | 24 | 61.4 | 37.5 | 240 | 82.7 |
|  | HRMEx27R2 | GAAGCTTTTGCACAAAATCAGATAGA | 26 | 61.8 | 34.6 |  |  |

*CFTR* (control gene) primers for GRACE-PCR

| Primer ID | Sequence | Size (bp) | Tm of Primer (°C) | % GC | Amplicon size (bp) | Tm of amplicon (°C) |
| --- | --- | --- | --- | --- | --- | --- |
| S2FP | TAAACCTCCCTGAAGAATCTTCC | 23 | 60.8 | 43.5 |  |  |
| S2RP | AGACCAGAGCAGGGACAGAA | 20 | 63.6 | 55 | 200 | 80 |
| S2RPD2 | TATGACCTAGGGAAATGGCTG | 21 | 60.3 | 47.6 | 250 | 78.7 |
