# Supplementary material for: RB1 screening of retinoblastoma patients in Sri Lanka using targeted next generation sequencing (NGS) and gene ratio analysis copy enumeration PCR (GRACE-PCR)
Source: BMC Med Genomics. 2023 Nov 6;16:279. doi: 10.1186/s12920-023-01721-6 (PMC10626775; doi:10.1186/s12920-023-01721-6)
Supplement: Supplementary file 2 — Additional file 2: Characteristics of RB patients selected for this study [file 12920_2023_1721_MOESM2_ESM.docx]

**Additional file 2.** Characteristics of RB patients selected for this study

| Patient ID | Sex | UL/BL | Age at diagnosis | Tumour grouping | Histopathology | Family history |
| --- | --- | --- | --- | --- | --- | --- |
| RB 1 | Female | UL | 11 months | B | Moderately differentiated RB | Nil |
| RB 2 | Female | UL | 2 years | E |  | Nil |
| RB 3 | Female | UL | 5 months | B |  | Nil |
| RB 4 | Male | UL | 8 months | B |  | Nil |
| RB 5 | Male | BL | 9 months | L-E, R-D | Compatible with regressed RB | Nil |
| RB 6 | Female | UL | 9 months | D |  | Nil |
| RB 7 | Male | BL | 7 months | R-D, L-A | Well differentiated RB | Nil |
| RB 8 | Female | BL | 3 years | R-D, L-E | Moderately differentiated RB | Nil |
| RB9 | Female | UL | 5 months | D |  | Nil |
| RB10 | Female | BL | 10 months | R D, L E | Well differentiated RB | Nil |
| RB11 | Female | BL | 2 months | R- E, L-D | RB with associated necrosis & Moderately differentiated RB | Nil |
| RB12 | Female | UL | 1 year 9 months | D |  | Nil |
| RB13 | Male | UL | 2 years | E | Moderately differentiated RB | Nil |
| RB14 | Female | UL | 4 years | E | Compatible with RB with uveal tract involvement and vitreous seeding | Nil |
| RB15 | Male | UL | 21 days | D |  | Nil |
| RB16 | Male | UL | 1 year 7 months | D |  | Nil |
| RB17 | Female | BL | 1 year 3 months | R-A, L-D |  | Nil |
| RB18 | Male | UL | 2 years | D | Poorly differentiated RB | Nil |
| RB19 | Male | UL | 1 year 5 months | B |  | Nil |
| RB20 | Female | UL | 1 year 7 months | D | Well differentiated RB | Nil |
| RB21 | Female | BL | 2 years | R-A, L-B | Well differentiated RB | Nil |
| RB22 | Male | UL | 1 year 1 month | E |  | Nil |
| RB23 | Male | UL | 4 months | E | Shows areas of tumour necrosis, gliosis and evidence of haemorrahage, no evidence of viable tumour. Optic nerve free of tumour | Nil |
| RB24 | Male | BL | 3 months | R-E, L-B |  | 1^st^ degree |
| RB25 | Female | UL | 3 years 2 months | E | R- Moderately differentiated RB | Nil |
| RB26 | Male | BL | 4 months | R-E, L-D | Suggestive of regressed Rb with vitreous seeding | Nil |
| RB27 | Female | UL | 8 months | R-E | Well differentiated RB | Nil |
| RB28 | Male | BL | 7 months | R-A, L-E | Compatible with RB showing extensive necrosis | Nil |
| RB29 | Female | BL | 6 months | R-E, L-B | Compatible with RB with vitreous seeding | Nil |
| RB30 | Male | UL | 5 months | E | Moderately differentiated RB with evidence of regression | Nil |
| RB31 | Female | UL | 3 years | E | Moderately Differentiated RB | Nil |
| RB32 | Male | UL | 5 years | E | Moderately differentiated RB | Nil |
| RB 33 | Male | UL | 1 year | D | Intact Right eye compatible with calcification, optic nerve free of tumour | Nil |
| RB 34 | Male | BL | 2 months | L-E, R-B | L-Moderately differentiated RB | Nil |
| RB 35 | Female | BL | 4 months | R-D, L-B | Moderately Differentiated RB | Nil |
| RB 36 | Female | UL | 6 years | E | Well differentiated RB | Nil |
| RB 37 | Male | BL | 3 years | R-D, L-A |  | Nil |
| RB 38 | Female | UL | 3 years | D | Moderately Differentiated RB | Nil |
| RB 39 | Female | BL | 5 months | R - B, L- E | L- Moderately differentiated RB | Nil |
| RB 40 | Male | BL | 6 months | R-D, L-A | Compatible with RB and extensive calcification | Nil |
| RB 41 | Female | UL | 11 months | D |  | Nil |
| RB 42 | Female | UL | 4 years | D | Compatible with RB with extensive necrosis | Nil |
| RB 43 | Female | UL | 3 years | E | Moderately differentiated RB with optic nerve involvement | Nil |
| RB 44 | Male | UL | 10 months | E | Compatible with necrosed RB | Nil |
| RB 45 | Male | BL | 1 year 1 month | R-D, L-E | Moderately differentiated RB | Nil |
| RB 46 | Male | UL | 3 years | E | Moderately differentiated RB | Nil |
| RB 47 | Male | UL | 1 year | E | Compatible with extensive necrosis | Nil |
| RB 48 | Female | UL | 1 year 6 months | D | Well differentiated RB | Nil |
| RB 49 | Female | BL | 9 months | R - E, L - D | Poorly differentiated RB with extensive calcification | Nil |

R: Right eye, L: Left eye, UL: Unilateral, BL: Bilateral
